# Unscented Kalman Filter-Trained Neural Networks for Slip Model Prediction

**DOI:** 10.1371/journal.pone.0158492

**Published:** 2016-07-28

**Authors:** Zhencai Li, Yang Wang, Zhen Liu

**Affiliations:** State Key Laboratory of Robotics and System, Harbin Institute of Technology, Harbin 150001, China; Heidelberg University, GERMANY

## Abstract

The purpose of this work is to investigate the accurate trajectory tracking control of a wheeled mobile robot (WMR) based on the slip model prediction. Generally, a nonholonomic WMR may increase the slippage risk, when traveling on outdoor unstructured terrain (such as longitudinal and lateral slippage of wheels). In order to control a WMR stably and accurately under the effect of slippage, an unscented Kalman filter and neural networks (NNs) are applied to estimate the slip model in real time. This method exploits the model approximating capabilities of nonlinear state–space NN, and the unscented Kalman filter is used to train NN’s weights online. The slip parameters can be estimated and used to predict the time series of deviation velocity, which can be used to compensate control inputs of a WMR. The results of numerical simulation show that the desired trajectory tracking control can be performed by predicting the nonlinear slip model.

## Introduction

In recent years, as wheeled mobile robots (WMRs) have been implemented more popularly in unstructured environments, motion control problems have received considerable attention in the automation field. The phenomenon of wheel slippage always exists in certain real-life motion tasks that have critical effects on the locomotion of mobile robots, which cannot be ignored for accurate tracking control.

A dynamic-model-based method is proposed to estimate the longitudinal wheel slip and to detect the immobilized conditions of WMRs traveling on outdoor terrain [1]. Slippage causes the imperfect rolling of wheels, and makes the accurate tracking control difficult when vehicles travel across off-road environments [2]. In order to attain higher performances, some researchers have studied the influence of slippage based on terramechanics analysis [3–5]. These methods have been proposed to identify the soil parameters for the wheel–ground interaction model off-line, which are helpful to study the motility of mobile robots. However, the aspects of wheel–ground interaction are needed for accurate models, which are neither well known nor easily measurable in realistic applications, because they do not adapt to varying terrain properties. In order to enhance the real-time control of mobile robots and control accuracy, rapid learning algorithms are required to be developed for identifying the slip model online. Some researchers have considered slip ratio estimation and compensation for tracked robots and WMRs in real time [6, 7]. For skid-steered WMR control in the presence of wheel skidding and slipping, a Kalman Filter-based estimation system is designed by combining the inertial measurements with centimeter accuracy RTK-GPS measurements to provide essential posture, velocities, and perturbation information [8]. The EKF-based positioning and wheel slip-estimation scheme are designed to enhance the precise tracking of velocity and posture [9]. These methods are required to establish accurate system models and observe real-time slip velocities; however, they are difficult to obtain in real applications.

NN is well suited to approximate the uncertain or nonlinear functions due to its online learning ability and nonlinear characteristics [10]. They have been successful to identify dynamical system models in actual applications [11–14]. However, the most continuous observations are to some extent contaminated by noise, which limits the function of many techniques of identification and prediction. Therefore, these observations should be filtered by some effective nonlinear filter methods, such as the extended Kalman filter (EKF) and the unscented Kalman filter (UKF) [15–18].

In this paper, the predictive slip model is developed to work for real trajectory tracking of a WMR using UKF-trained NN model, which includes the longitudinal, lateral and turning slip parameters. These slip parameters are applied to define the weights of the NN, and adjusted by the UKF. The control task is to accurately track the reference trajectory. The proposed method depends on more reliable posture residuals rather than measurements of velocity.

In this paper, a feedforward NN model and a nonlinear filtering method are introduced in Section 2. An unscented Kalman filter-based NN weight estimation is presented in Section 3. Section 4 presents the slip model prediction based on the UKF and NN. The simulation of trajectory tracking is presented and discussed in Section 5. Finally, Section 6 concludes the main contributions and results of this paper.

## NN Model and Nonlinear Filtering Method

### The feedforward NN model

**Fig 1:**
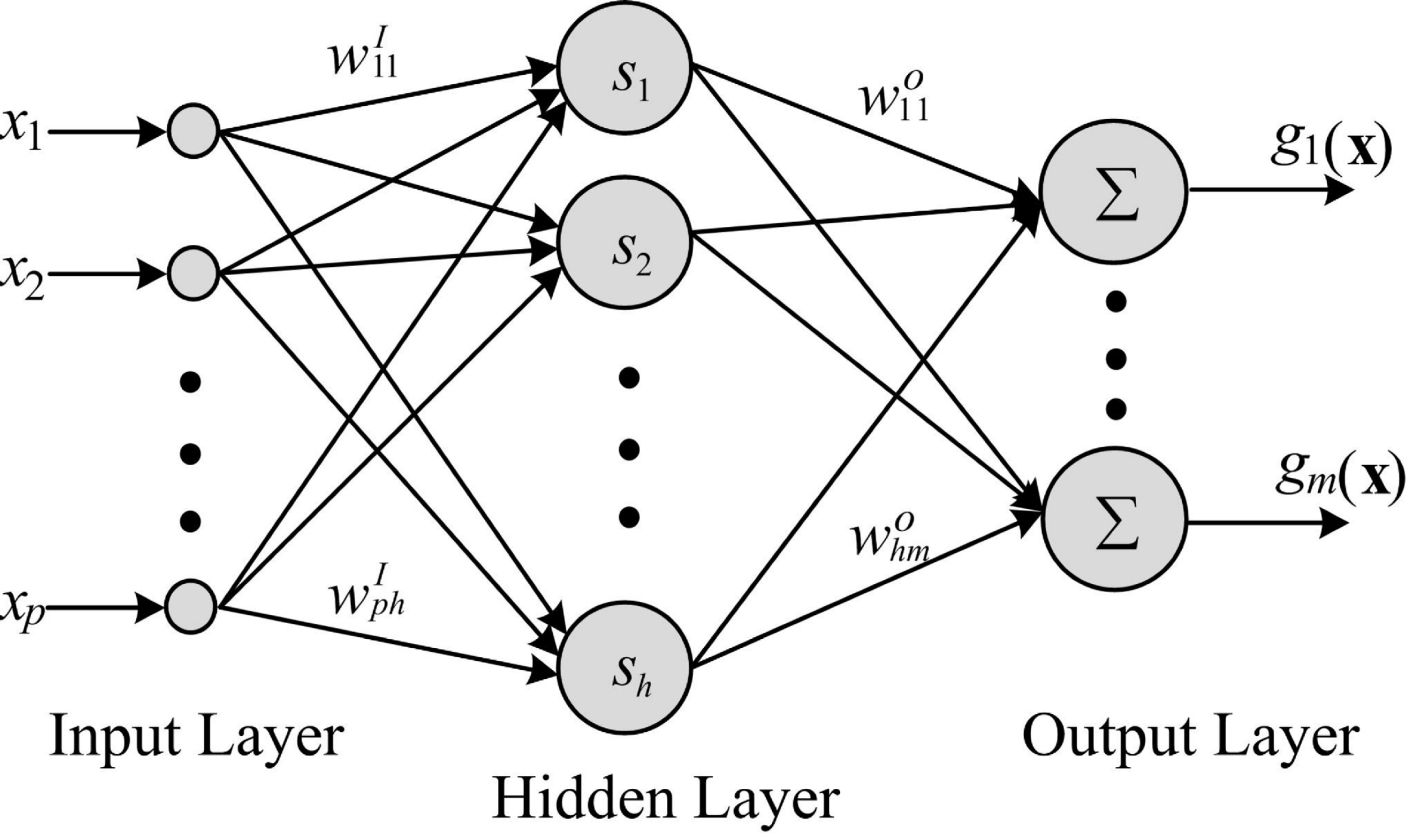
The structure of feedforward NN.

NNs have been widely applied to approximate nonlinear models due to their inherent learning capabilities [10–14]. A typical feedforward NN is applied in this paper, and its simple structure is shown in (Fig 1).

A nonlinear smooth function *g*(**x**):*R*^*p*^ → *R*^*m*^ can be expressed by the NN model:

$$s_{l}=s(\sum_{i=1}^{p}x_{i}w_{il}^{I}),\;l=1,\ldots ,h,$$ (1)

$$g_{j}(x)=\sum_{l=1}^{h}s_{l}w_{lj}^{o},\;j=1,\ldots ,m,$$ (2)

where **x** = [*x*_1_,…*x*_*p*_]^T^ is the input vector of an NN; **g** = [*g*_1_,…*g*_*m*_]^T^ is the output vector of an NN; matrix $w^{I}={\left\{w_{il}^{I}\right\}}_{p\times h}$ is the weight from input layer to the hidden layer; *s* is an activation function, and **s** = [*s*_1_,…,*s*_*h*_] is the output of the hidden layer; matrix $w^{o}={\left\{w_{lj}^{o}\right\}}_{h\times m}$ is the weight from the hidden layer to the output layer.

In this paper, a popular logistic sigmoid function is considered as the activation of hidden neuron for (1):

$$s(x)=\frac{1}{1+\exp(-x)}$$ (3)

## Nonlinear filtering method based feedforward NN training model

In this section, a filter is used to predict the state vector, which is composed of the NN weights. The model outputs of the NN are considered as the measurements of the nonlinear filter. In the nonlinear filter, the uncertainty in both states and measurements are correctly treated. The transition and observation models are used by standard nonlinear Kalman filtering algorithm as shown below.

### Transition Model

$$w_{k+1}=f(w_{k},x_{k})+\varepsilon_{k}$$ (4)

where $w_{k}={\left[w_{11}^{I}\ldots w_{1h}^{I},\ldots ,w_{p1}^{I}\ldots w_{ph}^{I},\;w_{11}^{o}\ldots w_{1m}^{o},\ldots ,\;w_{h1}^{o}\ldots w_{hm}^{o}\right]}_{k}$ is the weight vector in the *k*-th collected sample, and specifies the state transition equation corrupted by process noise **ε**_*k*_, which is characterized with covariance **Q**_*k*_.

### Observation Model

The output of the observation model can be expressed by

$$y_{k}=g(w_{k},x_{k})+\sigma_{k}$$ (5)

where **σ**_*k*_ is the observation noise, with the covariance **R**_*k*_,and ${\bar{y}}_{k}=g(w_{k},x_{k})$ denotes the predicted output.

### UKF-Based NN Weights Estimation

The unscented Kalman filter is an effective nonlinear filtering method [18–20], and has been widely applied in parameters prediction and estimation. The UKF–based NN weights estimation proceeds as follows, which is considered to predict the nonlinear model in the next section.

(a) Initialized with:
  $${\bar{w}}_{0}=E[w_{0}],\;P_{0}=E[(w_{0}-{\bar{w}}_{0}){\left(w_{0}-{\bar{w}}_{0}\right)}^{\text{T}}],$$ (6)
(b) Calculate the sigma points and weights for *k* ∈ {1,…,∞}:
  $$\chi_{0}=\bar{w};\;\chi_{i}=\bar{w}+{\left(\sqrt{(N+\lambda )P_{k}}\right)}_{i},\;i=1,\ldots ,N;$$ (7)
  $$\chi_{i}=\bar{w}-{\left(\sqrt{(N+\lambda )P_{k}}\right)}_{i-N},\;i=N+1,\ldots ,2N;$$ (8)
  $$W_{0}^{m}=\lambda /(N+\lambda );\;W_{0}^{c}=\lambda /(N+\lambda )+(1-a^{2}+b);$$ (9)
  $$W_{i}^{m}=W_{i}^{c}=1/2(N+\lambda ),\;i=1,\ldots ,2N;$$ (10)
  where $\bar{w}$ and **P**_*k*_ are assumed as the mean and covariance of **w**, respectively, *λ* = *a*^2^(*N* + *κ*) − *N* is the condition parameter, and parameter *a* decides the range of the sigma point around $\bar{w}$, which is often a small positive number (e.g., 1e–3). According to [19], we have *κ* = 0 and *b* = 2.

The vector of sigma points are mapped through the nonlinear function *g* to:

$$\gamma_{i}=g(\chi_{i}),\;i=0,\ldots ,2N.$$ (11)

According to the weighted posterior sigma points, the approximation of the mean and covariance for **y** can be obtained as:

$$\bar{y}\approx \sum_{i=0}^{2N}W_{i}^{\left(m\right)}\gamma_{i}$$ (12)

$$P_{yy}\approx \sum_{i=0}^{2N}W_{i}^{\left(c\right)}(\gamma_{i}-\bar{y}){\left(\gamma_{i}-\bar{y}\right)}^{\text{T}}$$ (13)

(c) Time update for prediction:
  $${\bar{\chi }}_{i,k}=f(\chi_{i,k-1},x_{k-1}),$$ (14)
  $${\bar{w}}_{k}=\sum_{i=0}^{2N}W_{i}^{\left(m\right)}{\bar{\chi }}_{i,k},$$ (15)
  $${\bar{P}}_{k}=\sum_{i=0}^{2N}W_{i}^{\left(c\right)}({\bar{\chi }}_{i,k}-{\bar{w}}_{k}){\left({\bar{\chi }}_{i,k}-{\bar{w}}_{k}\right)}^{\text{T}}+Q_{k},$$ (16)
  $${\bar{\gamma }}_{i,k}=g({\bar{\chi }}_{k},x_{k}),$$ (17)
  $${\bar{y}}_{k}=\sum_{i=0}^{2N}W_{i}^{\left(m\right)}{\bar{\gamma }}_{i,k}.$$ (18)
(d) Measurement update equations of the Gaussian statistics and observation:
  $$P_{y_{k}y_{k}}=\sum_{i=0}^{2N}W_{i}^{\left(c\right)}[{\bar{\gamma }}_{i,k}-{\bar{y}}_{k}]{\left[{\bar{\gamma }}_{i,k}-{\bar{y}}_{k}\right]}^{\text{T}}+R_{k},$$ (19)
  $$P_{w_{k}y_{k}}=\sum_{i=0}^{2N}W_{i}^{\left(c\right)}[{\bar{\chi }}_{i,k}-{\bar{w}}_{k}]{\left[{\bar{\gamma }}_{i,k}-{\bar{y}}_{k}\right]}^{\text{T}},$$ (20)
  $$K_{k}=P_{w_{k}y_{k}}P_{y_{k}y_{k}}^{-1},$$ (21)
  $${\hat{w}}_{k}={\bar{w}}_{k}+K_{k}(y_{k}-{\bar{y}}_{k}),$$ (22)
  $$P_{k}={\bar{P}}_{k}-K_{k}P_{y_{k}y_{k}}K_{k}^{\text{T}},$$ (23)
  $$P_{y_{k}}=\det{\left(2\pi P_{y_{k}y_{k}}\right)}^{-\frac{1}{2}}\exp[-\frac{1}{2}{\left(y_{k}-{\bar{y}}_{k}\right)}^{\text{T}}P_{y_{k}y_{k}}^{-1}(y_{k}-{\bar{y}}_{k})];$$ (24)
  where **Q**_*k*_ is the covariance of process noise, **R**_*k*_ is the covariance of measurement noise, and **K**_*k*_ is the Kalman gain.

### Slip Model Prediction of WMR

**Fig 2:**
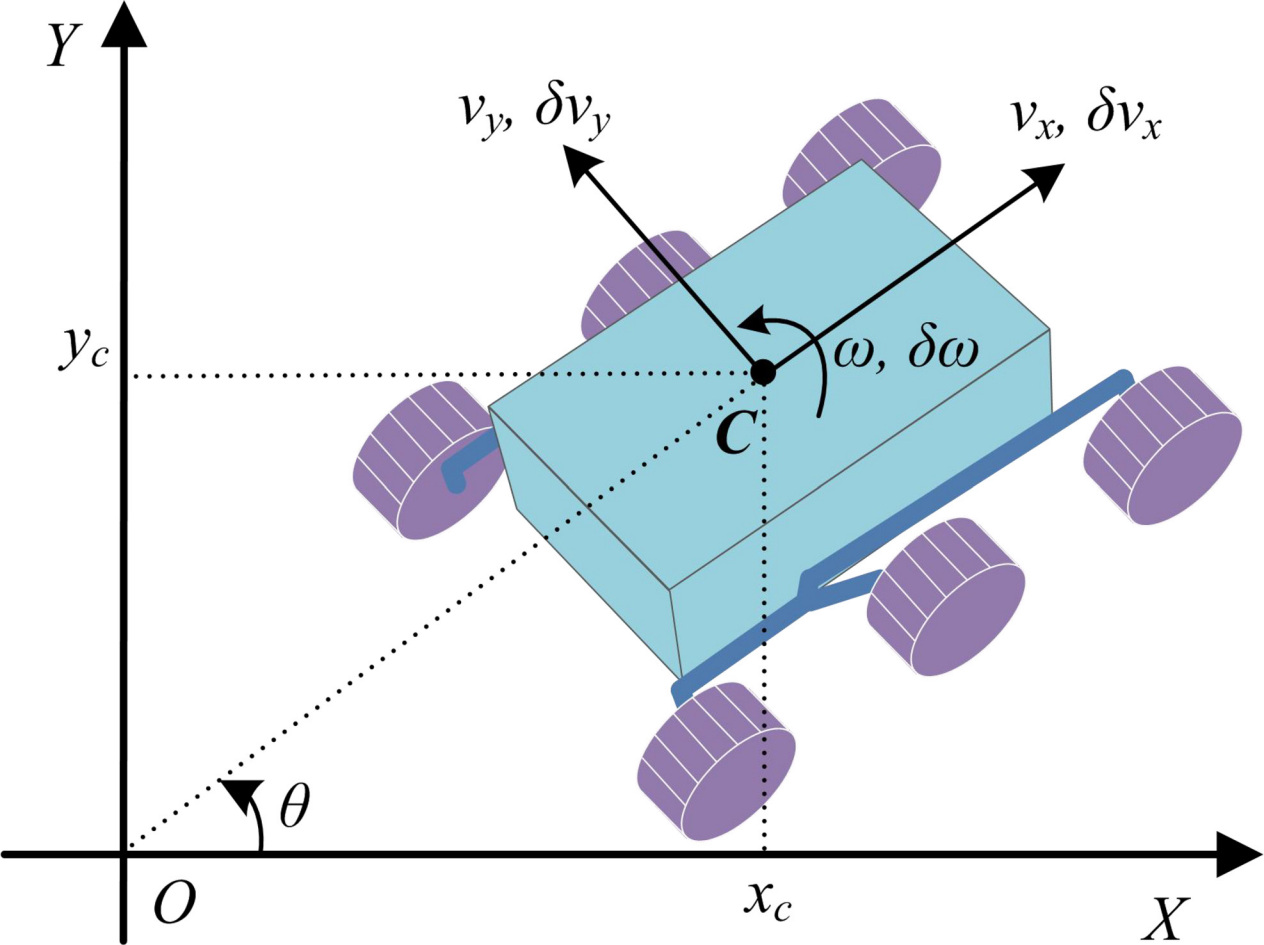
A six-wheel nonholonomic WMR.

The WMR is a typical nonholonomic mechanical system (Fig 2). The right and left wheels are driven independently. The motion and orientation are controlled by independent actuators, and torque of all wheels is provided by DC motors. For self-coordination of the robot, there are three degrees of freedom velocity including longitudinal, lateral, and turning angular velocities.

Generally, slippage usually happens between wheels and ground when a WMR is traveling in an outdoor environment. The kinematic differential equation of the WMR with respect to the world frame can be written by:

$$\dot{q}=\left[\begin{array}{c} {\dot{x}}_{c}\\ {\dot{y}}_{c}\\ \dot{\theta } \end{array}\right]=\left[\begin{array}{ccc} \cos\theta & -\sin\theta & 0\\ \sin\theta & \cos\theta & 0\\ 0 & 0 & 1 \end{array}\right]\left\{\left[\begin{array}{c} v_{x}\\ v_{y}\\ \omega \end{array}\right]+\left[\begin{array}{c} \delta v_{x}\\ \delta v_{y}\\ \delta \omega \end{array}\right]\right\}$$ (25)

or

$$\dot{q}=S(\theta )(u+\delta u)$$ (26)

where **u** = [*v*_*x*_ *v*_*y*_ *ω*]^T^ is the input vector of the kinematics model, which expresses a commanded component of velocity including the longitudinal, lateral, and turning angular velocities of WMR. Moreover, *δ***u** = [*δv*_*x*_(*α*_*x*_) *δv*_*y*_(*α*_*y*_) *δω*(*α*_*θ*_)]^T^ is the deviation of input vector, which expresses an error component of velocity including the forward slip ratio, *δv*_*x*_, the lateral slip ratio, *δv*_*y*_, and the angular slip ratio *δω*. Let **α** = [*α*_*x*_ *α*_*y*_ *α*_*θ*_]^T^ denote the uncertain slip parameters.

It is important to express the slip model in terms of control inputs rather than measurement, because the model will be used to predict the motion before traversing the terrain. Since the wheeled robot is influenced by the slippery terrain, the key calibration is how best to establish the slip model. Generally, the slip model can be expressed as a nonlinear function:

$$\dot{q}=h(q,u,\delta u(\alpha ))$$ (27)

The real trajectory of wheeled robot is obtained by integrating the Eq (27):

$$\begin{array}{l} q=\int h(q(t),u(t),\delta u(\alpha ))dt\\ \;=H(q,u,\alpha ,t) \end{array}$$ (28)

Any wheeled robot will depend on the terrain characteristics that are various over space and time due to some factors. Only real-time systems can adaptively change as fast as the local terrain, as a vehicle moves from one place to another. While the trajectory is followed, there is no clear method to show when the terrain will be changed. Therefore, the dynamic system must be able to be calibrated on any arbitrary trajectory.

It is obvious that the slip velocity is directly related to the input velocities *v*_*x*_, *v*_*y*_, and *ω*. The input lateral velocity is zero, *v*_*y*_ = 0, due to the nonholonomic character of the WMR. The number of inputs to the NN is *p* = 2. And clearly, the number of outputs is *m* = 3; here, we choose the number of the hidden layer neurons as *h* = 3. Therefore, the number of slip parameters is *N* = 15.

In order to obtain the accurate slip model (28), the NN model given in (2) can be recast by:

$$\delta u=\frac{1}{1+\exp(-xw^{I})}\cdot w^{o}$$ (29)

where the state vectors are defined as **x** = **u**, **w** = **α**. Each output of the NN can be expressed by the function of slip parameters as following:

$$\delta v_{x}=\sum_{l=1}^{3}s(\sum_{i=1}^{2}x_{i}w_{il}^{I})w_{l1}^{o}$$ (30)

$$\delta v_{y}=\sum_{l=1}^{3}s(\sum_{i=1}^{2}x_{i}w_{il}^{I})w_{l2}^{o}$$ (31)

$$\delta \omega =\sum_{l=1}^{3}s(\sum_{i=1}^{2}x_{i}w_{il}^{I})w_{l3}^{o}$$ (32)

where *x*_1_ = *v*_*x*_, *x*_2_ = *ω*; the slip parameters **α** = **w** can be expressed by three slip vectors as following:

$$w_{x}=[w_{11}^{I},w_{12}^{I},w_{13}^{I},w_{21}^{I},w_{22}^{I},w_{23}^{I},w_{11}^{o},w_{21}^{o},w_{31}^{o}]$$ (33)

$$w_{y}=[w_{11}^{I},w_{12}^{I},w_{13}^{I},w_{21}^{I},w_{22}^{I},w_{23}^{I},w_{12}^{o},w_{22}^{o},w_{32}^{o}]$$ (34)

$$w_{\omega }=[w_{11}^{I},w_{12}^{I},w_{13}^{I},w_{21}^{I},w_{22}^{I},w_{23}^{I},w_{13}^{o},w_{23}^{o},w_{33}^{o}]$$ (35)

In this paper, slip parameters are assumed to be constant over a short segment once the terrain and the inputs are known. The process noise is considered as Gaussian distribution in states observation. Therefore, the transition model (4) can be adequately obtained by:

$$w_{k+1}=w_{k}+\varepsilon_{k}.$$ (36)

According to the nonlinear integrated function (28), the observation model (5) can be obtained by:

$$y_{k}=H(y_{k-1},u_{k},w_{k})+\sigma_{k}.$$ (37)

### Simulation and Analysis of WMR Trajectory Tracking

The slip parameters of the WMR are the important state variables of tracking control, since wheel-ground slip will change the current velocity of the WMR and influence the tracking precision. We performed the numerical simulation to demonstrate the capabilities of the proposed method by collecting data on a six-wheeled skid-steered mobile robot. This simulation is used to imitate the actual environment, which includes wheel–ground interaction and wheel slip. For such a simulation, the execution of trajectory will create wheel slip, which causes the difficulty of tracking control based on the dynamics model. The predicted slip parameters can be used to estimate the error in trajectory prediction for planning purposes.

**Fig 3:**
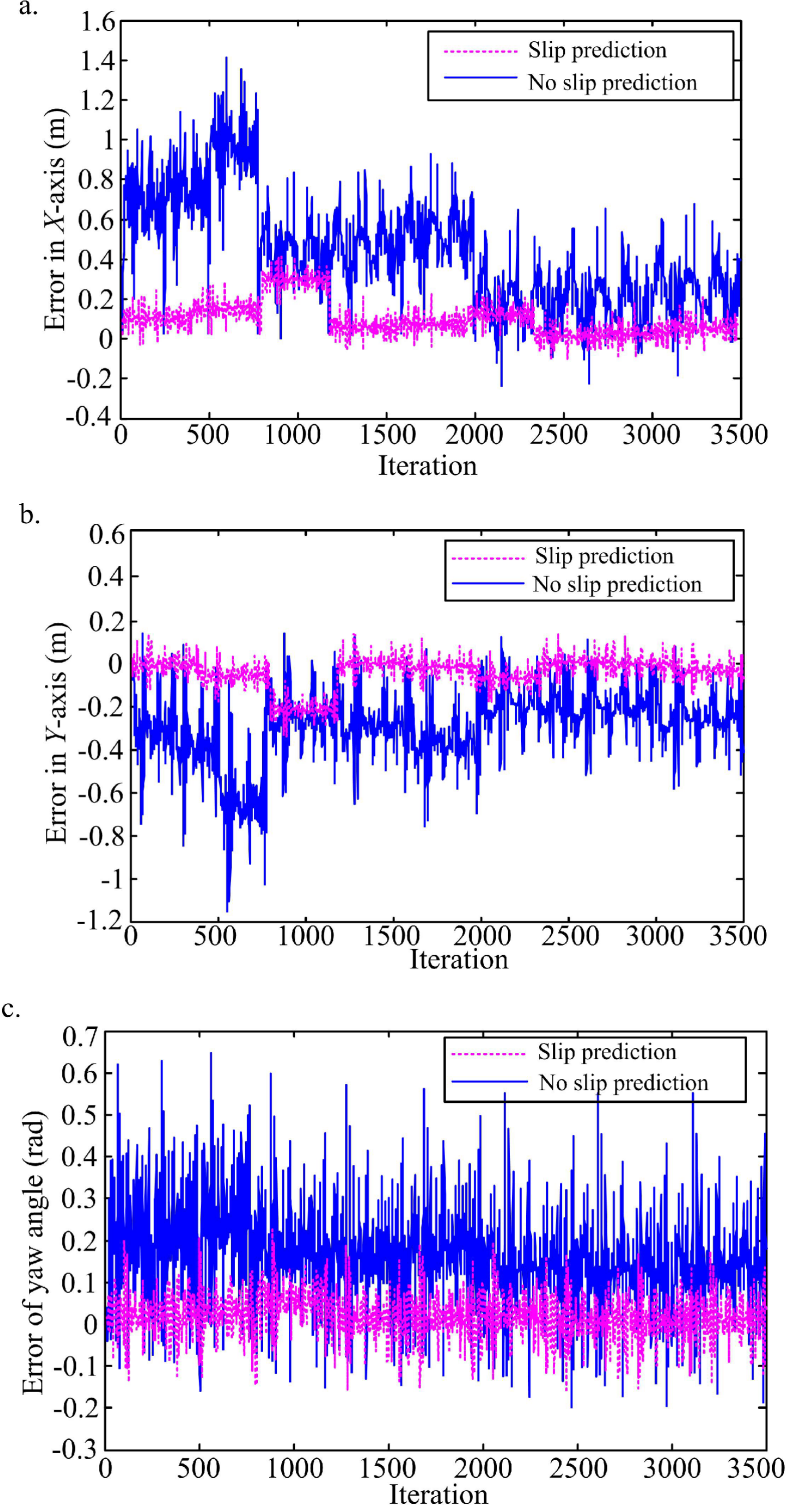
Comparison of tracking errors between slip prediction and no slip prediction. (a) Posture tracking error in *X*-axis. (b) Posture tracking error in *Y*-axis. (c) Tracking error of yaw angle.

In this simulation, a slippery flat terrain is considered for the WMR to travel, and three linear velocities of 0.1 m/s, 0.4 m/s, and 0.8 m/s, and three turning angular velocities of 0.1 rad/s, 0.2 rad/s, and 0.4 rad/s are commanded to drive on nine different curves. According to the collected data during the nine trajectories, the UKF is used to optimize the slip parameters of the kinematics model. As the UKF was run on the observed states, a following trajectory segment is predicted by estimating the current slip model from the current posture. The comparison of tracking errors between slip prediction and no slip prediction based on proportional derivative (PD) control scheme to track the same trajectories have presented in (Fig 3). According to the comparison, it is clear that the slip prediction is important for tracking control, which can reduce the posture errors of trajectory tracking. Therefore, the proposed method for slip parameters estimation and slip model prediction has the advantage of improving the accuracy of tracking control.

**Fig 4:**
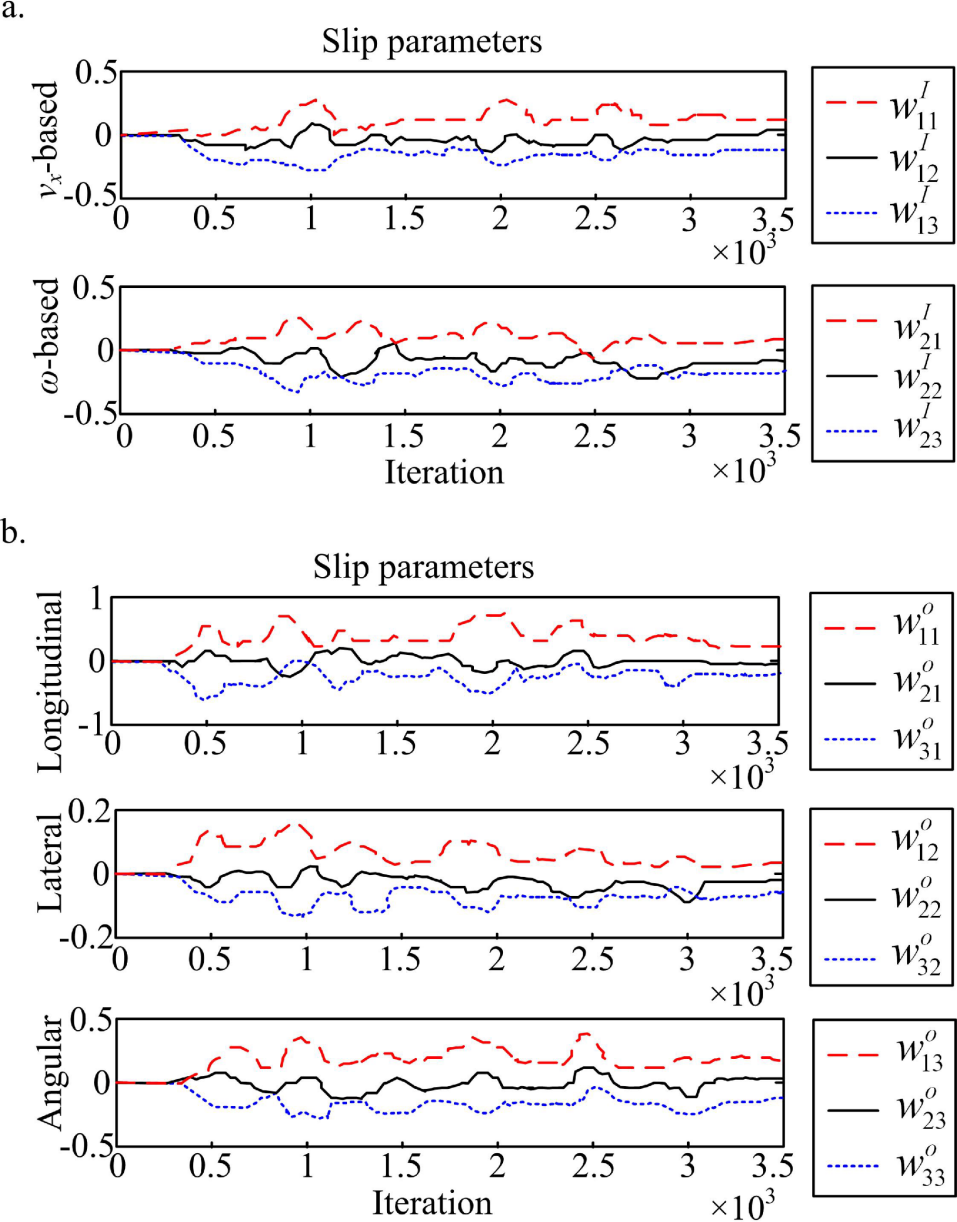
Slip parameters variation during UKF iterations. (a) NN weights **w**^*I*^. (b) NN weights **w**^*O*^.

It is significant that the slip surface parameters are all initialized to zero, and making no assumptions about the wheel-ground interaction. There is a rapid on-line adaptation to the different terrains. The slip parameters variation over the UKF run is shown in (Fig 4) versus the algorithm iteration index. When new operation terrains with different slip features are first encountered, the slip parameters can be clearly adjusted to obtain new knowledge.

**Fig 5:**
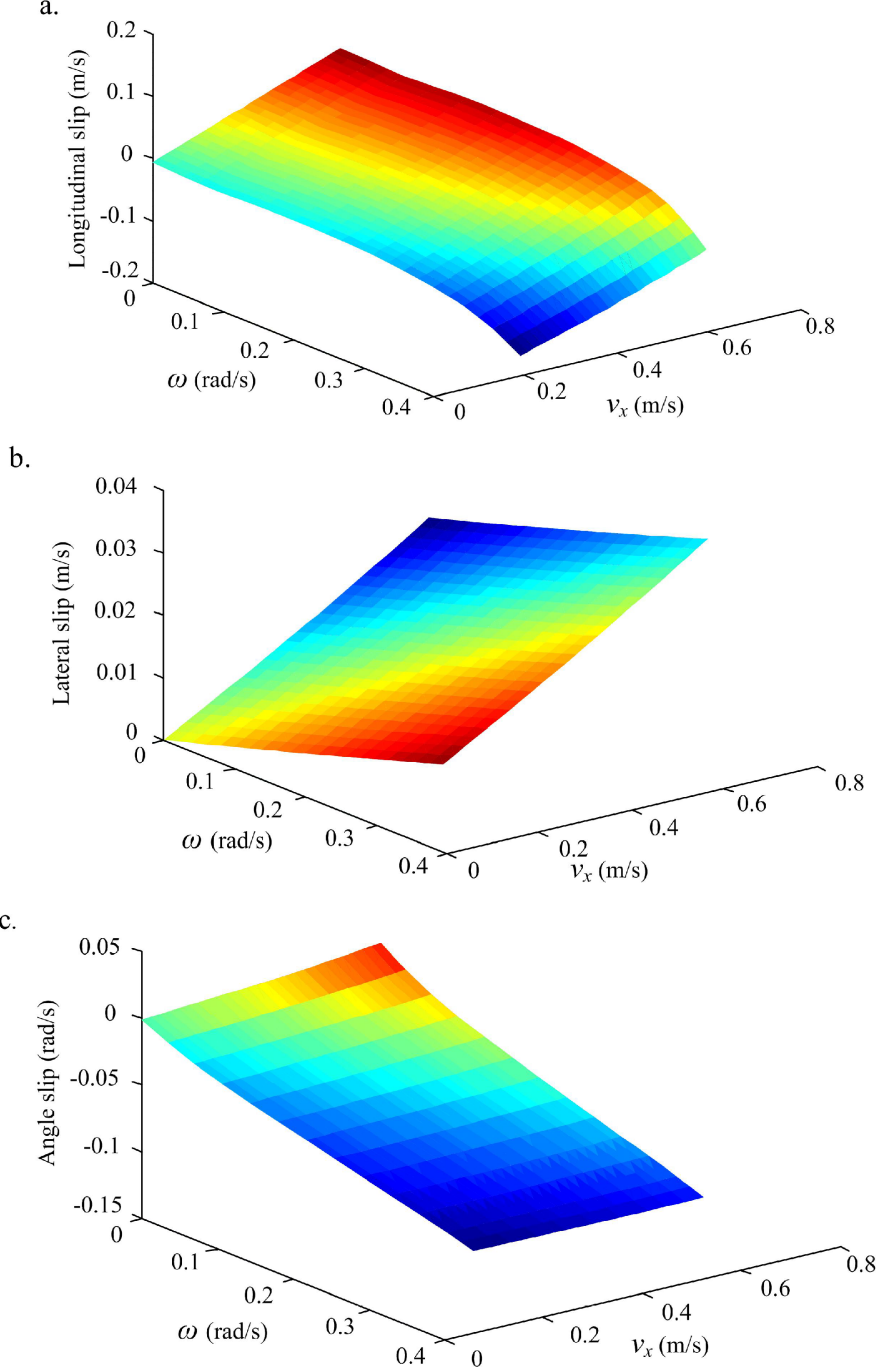
Slip Surfaces learned by UKF-based NN model training. (a) Longitudinal slip surface. (b) Lateral slip surface. (c) Angle slip surface.

The final slip surfaces “*δv*_*x*_,*δv*_*y*_, and *δω*” for the six-wheel mobile robot can be obtained on this terrain as shown in (Fig 5). These surfaces are the final predicted slip rates over the observed command forward and rotational velocities. The slip surfaces show that the changing range of lateral slip rate is very small due to the nonholonomic character of the WMR and traveling on the flat terrain; the rotational and forward slip, however, have the dominant effect due to the longitudinal wheels slip.

In order to validate the importance of the predictive slip model and test its generalizability, WMR is applied to track four different trajectories, where the white noises at different levels are added into the states observation. Since lateral velocity input can not be commanded due to WMR’s nonholonomic feature. Therefore, in order to realize the trajectory tracking control in a high accuracy, and ensure the longitudinal and lateral position errors minimization, the error of yaw angle has to be sacrificed. In the following, four different trajectories tracking control will be performed.

**Fig 6:**
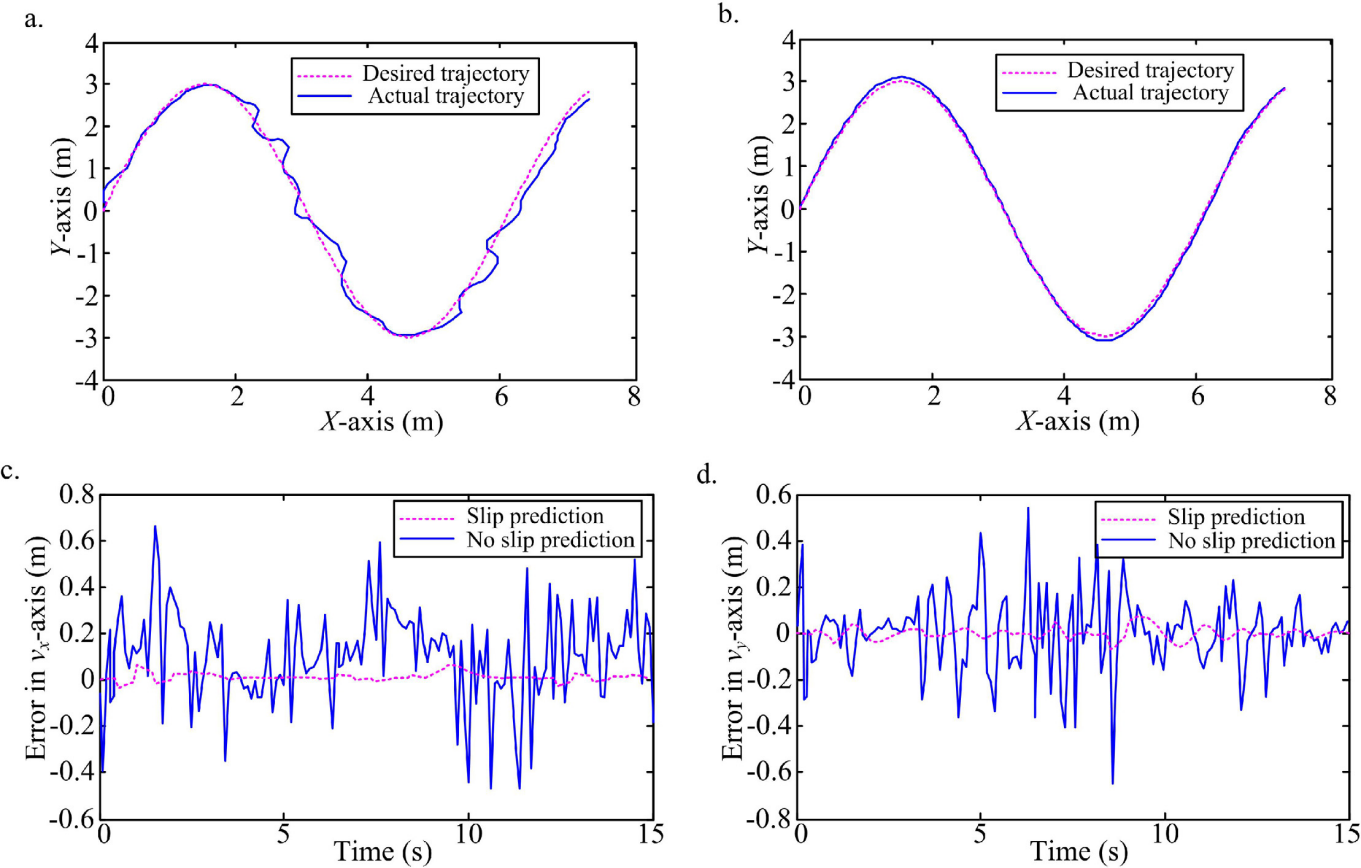
Tracking a sinusoidal with slip prediction compared to the desired trajectory and ideal trajectory without slip prediction. (a) Trajectory tracking without slip prediction and the desired trajectory. (b) Trajectory tracking with slip prediction and the desired trajectory. (c) Tracking errors with slip prediction and no slip prediction in *v*_*x*_-axis. (d) Tracking errors with slip prediction and no slip prediction in *v*_*y*_-axis.

**Fig 7:**
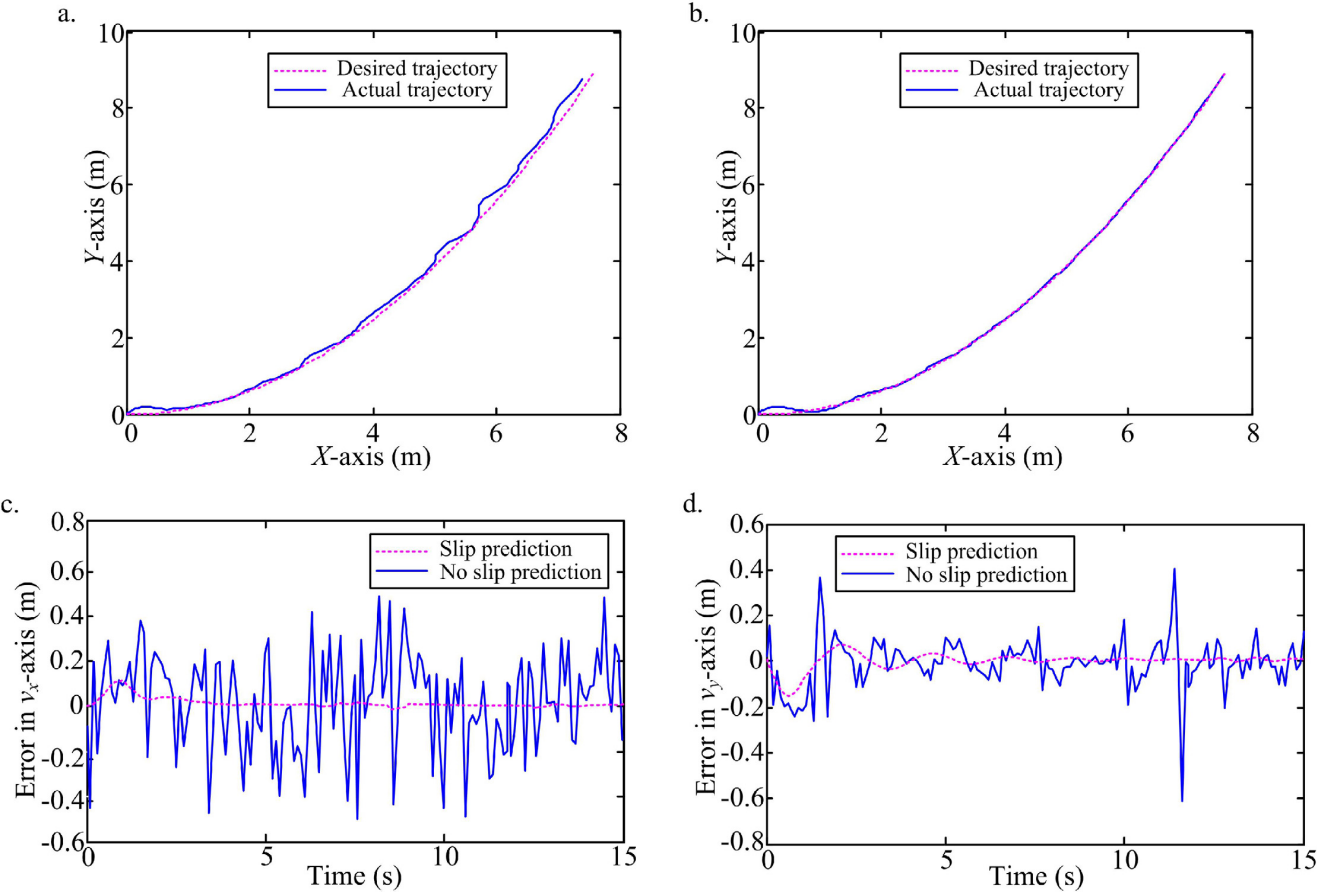
Tracking a parabola with slip prediction compared to the desired trajectory and ideal trajectory without slip prediction. (a) Trajectory tracking without slip prediction and the desired trajectory. (b) Trajectory tracking with slip prediction and the desired trajectory. (c) Tracking errors with slip prediction and no slip prediction in *v*_*x*_-axis. (d) Tracking errors with slip prediction and no slip prediction in *v*_*y*_-axis.

Firstly, a low level white noise in the interval [-0.4, 0.6] is added into the states observation. A sinusoidal (Fig 6, S1 Table) and a parabola (Fig 7, S2 Table) trajectories are tracked under the white noise. According to the comparison of trajectories tracking between no slip prediction and slip prediction, and the comparison of tracking errors in the local coordinate of WMR, it is obvious that the actual trajectories with slip prediction are closer the desired ones than trajectories without slip prediction. Clearly the predictive slip model for this robot plays an important role for tracking a trajectory on the slippery terrain.

**Fig 8:**
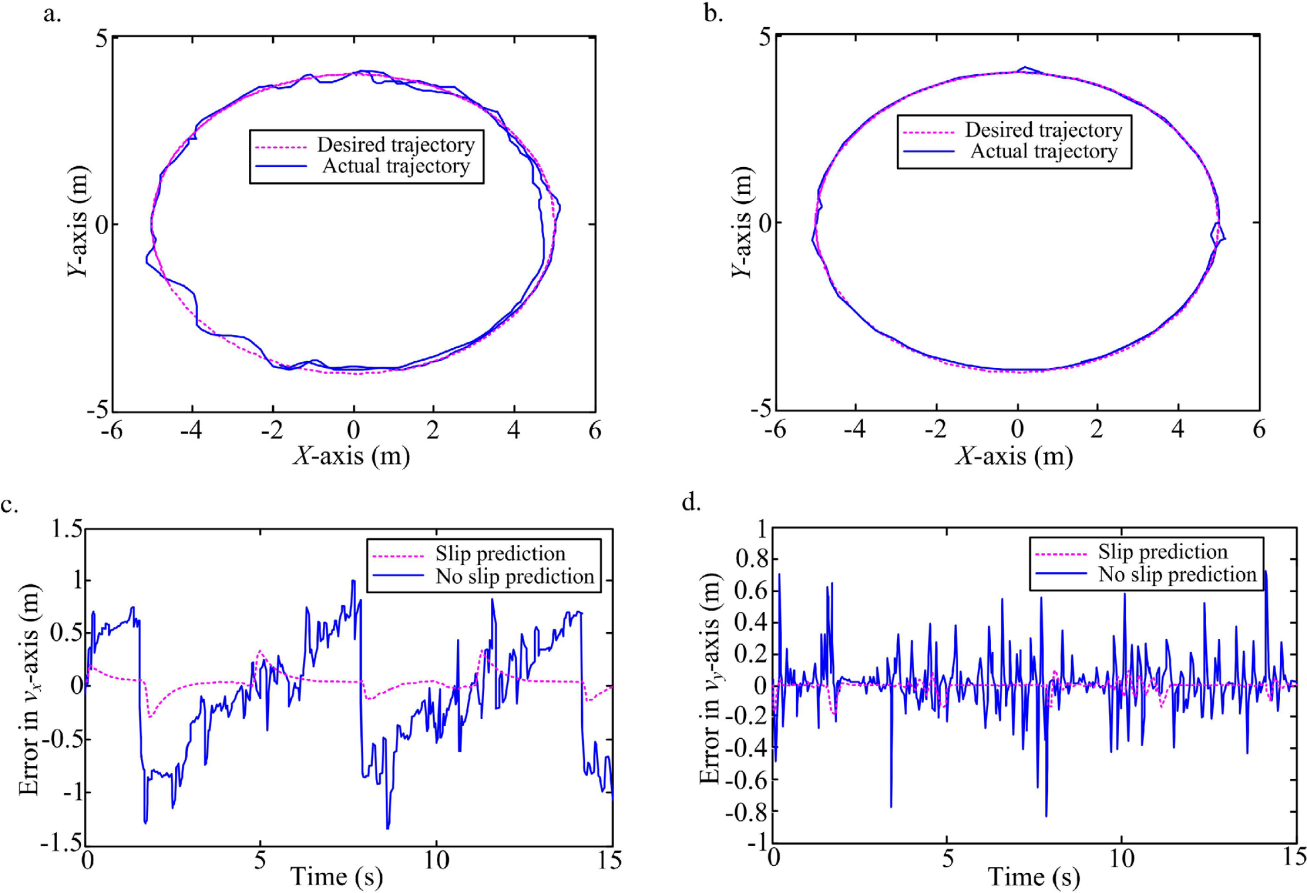
Tracking an ellipse with slip prediction compared to the desired trajectory and ideal trajectory without slip prediction. (a) Trajectory tracking without slip prediction and the desired trajectory. (b) Trajectory tracking with slip prediction and the desired trajectory. (c) Tracking errors with slip prediction and no slip prediction in *v*_*x*_-axis. (d) Tracking errors with slip prediction and no slip prediction in *v*_*y*_-axis.

**Fig 9:**
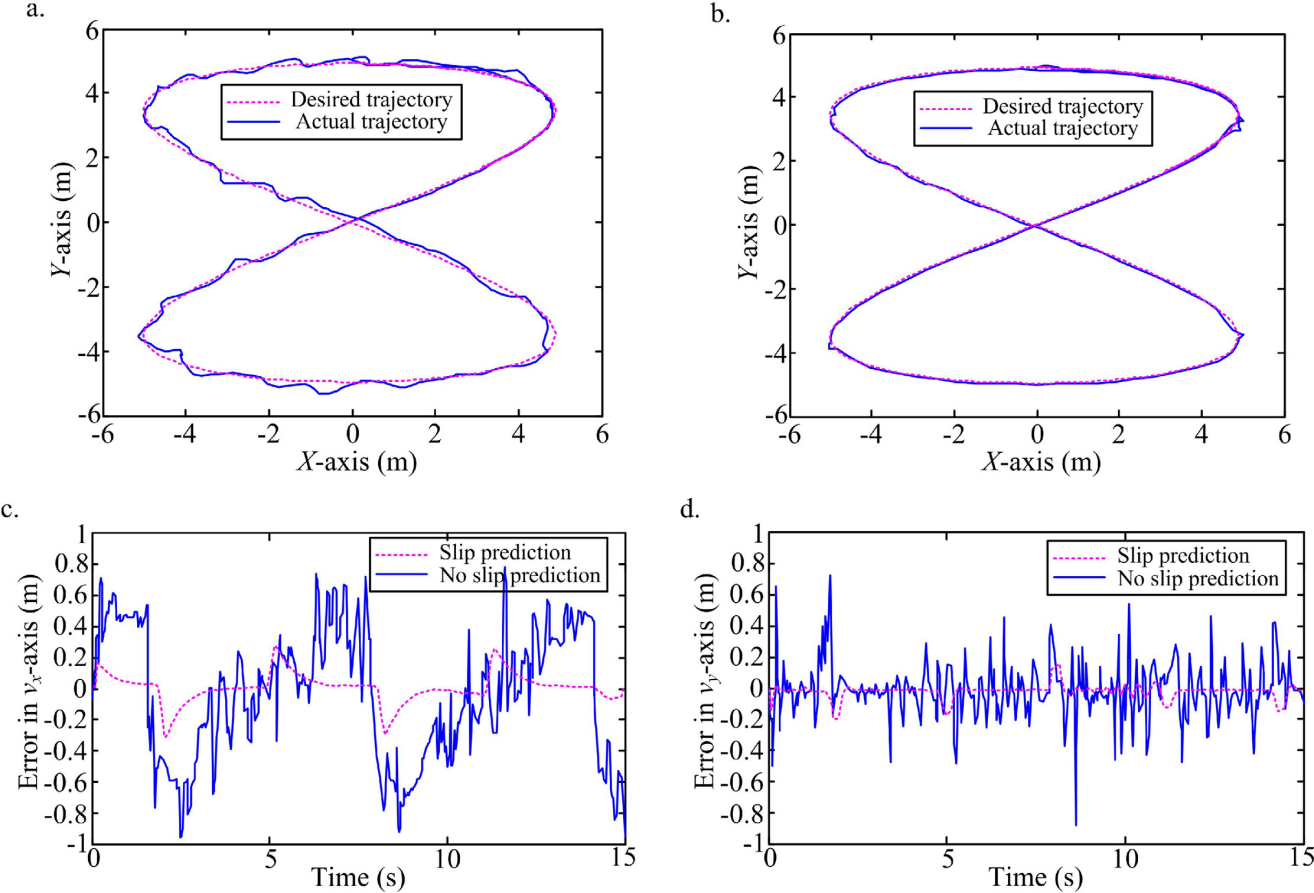
Tracking an 8-curve with slip prediction compared to the desired trajectory and ideal trajectory without slip prediction. (a) Trajectory tracking without slip prediction and the desired trajectory. (b) Trajectory tracking with slip prediction and the desired trajectory. (c) Tracking errors with slip prediction and no slip prediction in *v*_*x*_-axis. (d) Tracking errors with slip prediction and no slip prediction in *v*_*y*_-axis.

In order to further confirm the model's predictive power, a high-level white noise in the interval [-3, 3] is added into the states observation. An ellipse (Fig 8, S3 Table) and an 8-curve (Fig 9, S4 Table) trajectories are tracked under the high-level white noise. According to the comparison of trajectories tracking between no slip prediction and slip prediction, and the comparison of tracking errors in the local coordinate of WMR, it is obvious that the actual trajectories with slip prediction are closer the desired ones than trajectories without slip prediction. Clearly, though the high level noise is considered, the slip prediction can still perform the trajectory tracking with high accuracy on the slippery terrain.

## Conclusions

In this paper, an effective method of parameters estimation is developed to predict WMR’s slip model rapidly. The UKF-based NN model learning is applied to predict slip parameters online. The weights of the NN are applied in the state model of the UKF and the output of the NN presents the deviation model, and the observation model of the UKF is the integrating form of the kinematics model. The method applies the slip model to express the phenomenon of slippage, and slip velocities are related to arbitrary input signals of the model. We have demonstrated nine circle trajectories with different velocities for tracking on the slippery terrain, and presented the posture residuals based on the predictive slip parameters. The method is clearly successful over all the input space for our six-wheel mobile robot and is suitable to apply in different slippery terrains. Furthermore, the simulations of tracking four different trajectories are performed with white noises at different levels. The results have shown that the proposed method for slip prediction can improve the tracking accuracy for the desired trajectories and adapting the slippery terrains rapidly.

## Supporting Information

S1 Table List of positions and tracking errors under traveling a sinusoidal without slip prediction and with slip prediction. Column description: Xr, Yr: the desired positions in X-axis and Y-axis. X no SP: the actual position without slip prediction in X-axis. Y no SP: the actual position without slip prediction in Y-axis. xe no SP: the position error without slip prediction in *v*_*x*_-axis. ye no SP: the position error without slip prediction in *v*_*y*_-axis. Noise: the white noise with low level. X with SP: the actual position with slip prediction in X-axis. Y with SP: the actual position with slip prediction in Y-axis. xe with SP: the position error with slip prediction in *v*_*x*_-axis. ye with SP: the position error with slip prediction in *v*_*y*_-axis.
(XLS)

S2 Table List of positions and tracking errors under traveling a parabola without slip prediction and with slip prediction. Column description: Xr, Yr: the desired positions in X-axis and Y-axis. X no SP: the actual position without slip prediction in X-axis. Y no SP: the actual position without slip prediction in Y-axis. xe no SP: the position error without slip prediction in *v*_*x*_-axis. ye no SP: the position error without slip prediction in *v*_*y*_-axis. X with SP: the actual position with slip prediction in X-axis. Y with SP: the actual position with slip prediction in Y-axis. xe with SP: the position error with slip prediction in *v*_*x*_-axis. ye with SP: the position error with slip prediction in *v*_*y*_-axis.
(XLS)

S3 Table List of positions and tracking errors under traveling an ellipse without slip prediction and with slip prediction. Column description: Xr, Yr: the desired positions in X-axis and Y-axis. X no SP: the actual position without slip prediction in X-axis. Y no SP: the actual position without slip prediction in Y-axis. xe no SP: the position error without slip prediction in *v*_*x*_-axis. ye no SP: the position error without slip prediction in *v*_*y*_-axis. Noise: the white noise with high level. X with SP: the actual position with slip prediction in X-axis. Y with SP: the actual position with slip prediction in Y-axis. xe with SP: the position error with slip prediction in *v*_*x*_-axis. ye with SP: the position error with slip prediction in *v*_*y*_-axis.
(XLS)

S4 Table List of positions and tracking errors under traveling an 8-curve without slip prediction and with slip prediction. Column description: Xr, Yr: the desired positions in X-axis and Y-axis. X no SP: the actual position without slip prediction in X-axis. Y no SP: the actual position without slip prediction in Y-axis. xe no SP: the position error without slip prediction in *v*_*x*_-axis. ye no SP: the position error without slip prediction in *v*_*y*_-axis. X with SP: the actual position with slip prediction in X-axis. Y with SP: the actual position with slip prediction in Y-axis. xe with SP: the position error with slip prediction in *v*_*x*_-axis. ye with SP: the position error with slip prediction in *v*_*y*_-axis.
(XLS)
